# A High Burden of Hypertension in the Urban Black Population of Cape Town: The Cardiovascular Risk in Black South Africans (CRIBSA) Study

**DOI:** 10.1371/journal.pone.0078567

**Published:** 2013-11-08

**Authors:** Nasheeta Peer, Krisela Steyn, Carl Lombard, Nomonde Gwebushe, Naomi Levitt

**Affiliations:** 1 Chronic Diseases of Lifestyle Unit, Medical Research Council, Durban, South Africa; 2 Chronic Disease Initiative for Africa, Department of Medicine, University of Cape Town, Cape Town, South Africa; 3 Biostatistics Unit, Medical Research Council, Cape Town, South Africa; 4 Division of Endocrinology and Diabetes, Department of Medicine, Cape Town, South Africa; Universidad Peruana de Ciencias Aplicadas (UPC), Peru

## Abstract

**Objective:**

To determine the prevalence, associations and management of hypertension in the 25–74-year-old urban black population of Cape Town and examine the change between 1990 and 2008/09 in 25–64-year-olds.

**Methods:**

In 2008/09, a representative cross-sectional sample, stratified for age and sex, was randomly selected from the same townships sampled in 1990. Cardiovascular disease risk factors were determined by administered questionnaires, clinical measurements and fasting biochemical analyses. Logistic regression models evaluated the associations with hypertension.

**Results:**

There were 1099 participants, 392 men and 707 women (response rate 86%) in 2008/09. Age-standardised hypertension prevalence was 38.9% (95% confidence interval (CI): 35.6–42.3) with similar rates in men and women. Among 25–64-year-olds, hypertension prevalence was significantly higher in 2008/09 (35.6%, 95% CI: 32.3–39.0) than in 1990 (21.6%, 95% CI: 18.6–24.9). In 2008/09, hypertension odds increased with older age, family history of hypertension, higher body mass index, problematic alcohol intake, physical inactivity and urbanisation. Among hypertensive participants, significantly more women than men were detected (69.5% vs. 32.7%), treated (55.7% vs. 21.9%) and controlled (32.4% vs. 10.4%) in 2008/09. There were minimal changes from 1990 except for improved control in 25–64-year-old women (1990∶14.1% vs. 2008/09∶31.5%).

**Conclusions:**

The high and rising hypertension burden in this population, its association with modifiable risk factors and the sub-optimal care provided highlight the urgent need to prioritise hypertension management. Innovative solutions with efficient and cost-effective healthcare delivery as well as population-based strategies are required.

## Introduction

Hypertension is regarded as one of Sub-Saharan Africa’s (SSA) greatest health challenges after HIV/AIDS [1]; a far cry from the early 20^th^ century when hypertension was rare in the region [2]. Furthermore, unlike high-income countries where mean blood pressure (BP) has decreased over the last three decades, in Africa it has remained stable or increased in most countries [3] with hypertension emerging as the most prevalent cardiovascular disease (CVD) risk factor in the latter half of the 20^th^ century [2].

In South Africa, high BP contributes substantially to the CVD burden and in 2000, after sexually transmitted diseases, was the second leading risk factor contributing to mortality in the country [4]. Almost 47 000 deaths or 9% of total mortality was attributable to high BP indicating the profound effect of this disease burden on the local population. Approximately 50% of stroke, 42% of IHD and 22% of other CVD burden in ≥30-year-old adults was on account of high BP [4].

Nonetheless, there is a dearth of national hypertension surveillance data with the most recent conducted in 2003, and on a regional level these have not been ascertained in the urban black population of Cape Town in almost two decades. It is imperative, notwithstanding, to establish the prevalence, distribution, management and trends of hypertension in a population in order to appropriately allocated resources and develop cost-effective therapeutic strategies and programmes. Given the suggestion that the prevalence of hypertension is rising in SSA, together with urbanisation and changes in demography with ageing populations in South Africa [5], it is reasonable to assume that the prevalence of hypertension has also increased in the urban black population of Cape Town.

Therefore, the Cardiovascular Risk in Black South Africans (CRIBSA) study aimed to ascertain the prevalence and quality of care of hypertension as well as its associated factors in the urban black population of Cape Town and to compare these findings with a similar study conducted in 1990.

## Materials and Methods

### Study Population and Sampling Procedure

A random sample of 25–74-year-old men and women in the predominantly black residential areas of Langa, Guguletu, Crossroads, Nyanga and Khayelitsha in Cape Town in 2008/09 participated in this cross-sectional study. These areas were selected to ensure comparability with a 1990 study, the methodology of which has been previously described [6]. Since the hypertension prevalence was historically much higher than the diabetes mellitus prevalence in this population, the sample size was planned based on an estimated diabetes mellitus prevalence of 8% with a precision of 1.5% two-sided with 95% confidence. The sampling procedure, described previously in detail, was done using aerial maps and comprised a 3-stage cluster sampling stratified by area and housing type [7]. Random sampling of residential blocks within the main strata (stage 1) was followed by systematic sampling of plots, flats or structures within blocks (stage 2). Thereafter, individuals from households were selected using quotas (stage 3), pre-specified by age and gender categories, and disproportionate across age groups to ensure at least 50 men and women in each sex category [7].

### Data Collection

Questionnaires, administered by trained fieldworkers, collected socio-demographic data, self-reported family and medical history, physical activity patterns (Global Physical Activity Questionnaire (GPAQ)) [8], psychosocial stress, tobacco (WHO STEP-wise surveillance questionnaire) [9] and alcohol use. The CAGE set of four questions, previously validated and used extensively in South Africa [10], assessed problematic alcohol use [11]. Ownership of consumer items (durable goods), dwelling characteristics in terms of wall and flooring materials, and the source of drinking water and toilet facilities categorised wealth by the asset index. The following tools examined psychosocial stress: 1) Brugha life events questionnaire comprised of 12 questions related to negative life events such as illness, death, financial or marital difficulties, etc. and their impact) [12], 2) Antonovsky’s sense of coherence (SOC) measured 13 items on comprehensibility (cognitive), manageability (instrumental/behavioural) and meaningfulness (motivational)) [13], where a low SOC inferred a low ability to cope with stressors [14], and 3) locus of control (LOC) (six questions) determined the individual’s perceived sense of control over his/her environment and life with a low score construing poor perceived control and a high score good perceived control [15].

Of the three blood pressure (BP) measurements taken at two-minute intervals using an Omron BP monitor with an appropriately sized cuff after the participant had been seated for five minutes, the average of the second and third BP measurements was used in the analysis. Standardised techniques measured height, weight, and waist and hip circumferences [16]. After an overnight fast of 10 hours, blood samples for glucose and lipid estimations were drawn, followed by a standard oral glucose tolerance test with blood samples taken 120 minutes later [17].

### Definitions

BP≥140/90 mmHg or using antihypertensive agents defined hypertension. The 1998 WHO definition diagnosed diabetes mellitus, impaired glucose tolerance (IGT) and impaired fasting glucose (IFG) [17]. The following defined dyslipidaemia [18]: total cholesterol (TC) >5 mmol/l, triglycerides >1.5 mmol/l, high density lipoprotein cholesterol (HDL-C) <1.2 mmol/l and calculated low density lipoprotein cholesterol (LDL-C) using the Friedewald equation >3.0 mmol/l [19] and HDL-C/TC ratio <20%.

Standardised international criteria identified normal weight, overweight and obesity, raised waist circumference (>94 cm in men and >80 cm and in women), and raised waist-to-hip ratio (WHR) (>1.0 in men and >0.85 in women) [20]. Smoking ≥1 cigarette a day defined smoking status. Two or more CAGE questions answered affirmatively deemed problematic alcohol use present [11]. Less than 150 minutes of moderate-to-vigorous activity per week defined physical inactivity.

### Statistical Analysis

STATA 11 [21] and SAS Version 9.2.3 [22] were used for data analyses. Descriptive statistics, including crude prevalence, were calculated using the weights based on the sample design and adjusted for the realised sample. A principal component analysis of the pooled data, based on the assets that defined wealth, was used to develop an asset index [23]. Categories of relative wealth were created using tertiles. The age-standardised hypertension prevalence was calculated using the WHO World Population as the standard [24]. Univariate analyses (socio-demographic and CVD risk characteristics) are presented as mean values and standard deviations for continuous data, and as percentages for categorical data.

The comparison between the 1990 and 2008/09 data was done for 25–64-year-olds because these were the common age categories between the two studies. A direct comparison of the 2008/09 and 1990 datasets could not be conducted because of the geographic and demographic changes that occurred in the residential areas of Khayelitsha and Langa during this period; the population increased markedly with a concomitant expansion in area size. This necessitated the use of 95% confidence intervals (CI) for the comparison of the hypertension data between the two surveys.

Survey multiple logistic regression analysis determined the independent associations of hypertension with the psychosocial factors, adjusting for a set of modifiable (physical inactivity, body mass index (BMI)) and non-modifiable (family history, age, sex and urbanisation) risk factors for hypertension. Variable cut-points for age, physical activity and urbanisation were selected based on the smoothed values shown in the fractional polynomials. The three psychosocial measures were modelled independently as continuous variables. A positive history of current smoking and problem drinking were included in all three models.

The University of Cape Town’s Research and Ethics Committee approved the study. All participants signed informed consent.

## Results

After excluding 17 participants because they did not meet the inclusion criteria, the realised sample comprised 1099 participants with 392 men and 707 women (64% and 108% of the planned sample, respectively). Among those invited to participate in the study, 84% of men and 87% of women accepted. Of the 187 non-responders (i.e. the selected individuals who the study team were unsuccessful in contacting), 79 (42%) were men.

**Figure 1:**
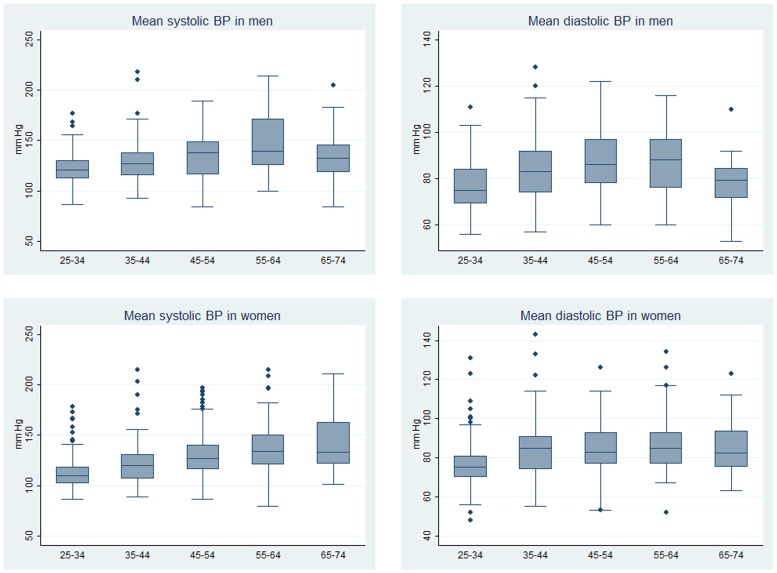
Mean systolic and diastolic blood pressures (mmHg) in men and women by age category (years).

**Figure 2:**
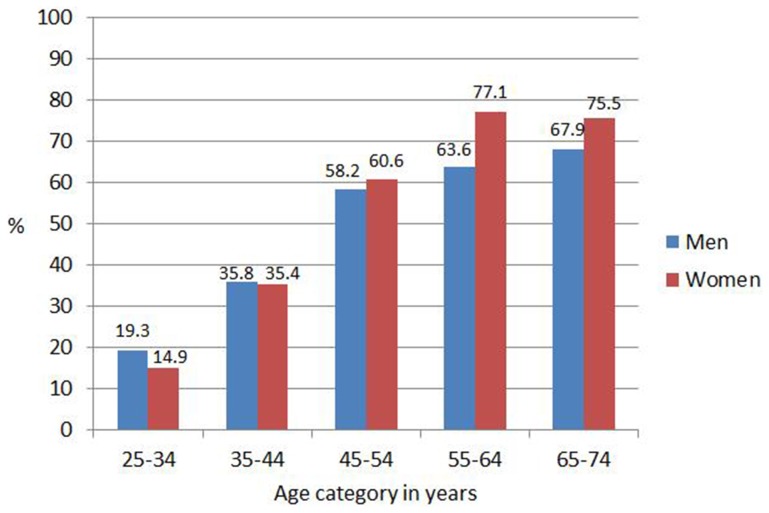
Prevalence of hypertension in 25–74-year-old men and women in 2008/09 presented by age category.

Mean systolic BP was higher in men (129.7 mmHg, 95% CI: 127.2–132.2) than women (121.6 mmHg, 95% CI: 119.6–123.5) (p<0.001) but their diastolic BP were similar (81.4 mmHg, 95% CI: 79.7–83.2 vs. 81.1 mmHg, 95% CI: 79.9–82.3, p = 0.750) **(** **Figure 1** **)**. The crude and age-standardised prevalence of hypertension was 37.5% (95% CI: 34.2–40.9) and 38.9%, (95% CI: 35.6–42.3), respectively, with similar rates in men (37.6%, 95% CI: 31.8–43.7 and 38.7%, 95% CI: 33.0–44.8) and women (37.5%, 95% CI: 33.7–41.4 and 39.0%, 95% CI: 35.2–43.0). There was a sharp increase in hypertension prevalence in ≥45-year-old men and women with the peak age by sex in 65–74-year-old men (67.9%) and in 55–64-year-old women (77.1%) **(** **Figure 2** **)**.

**Figure 3:**
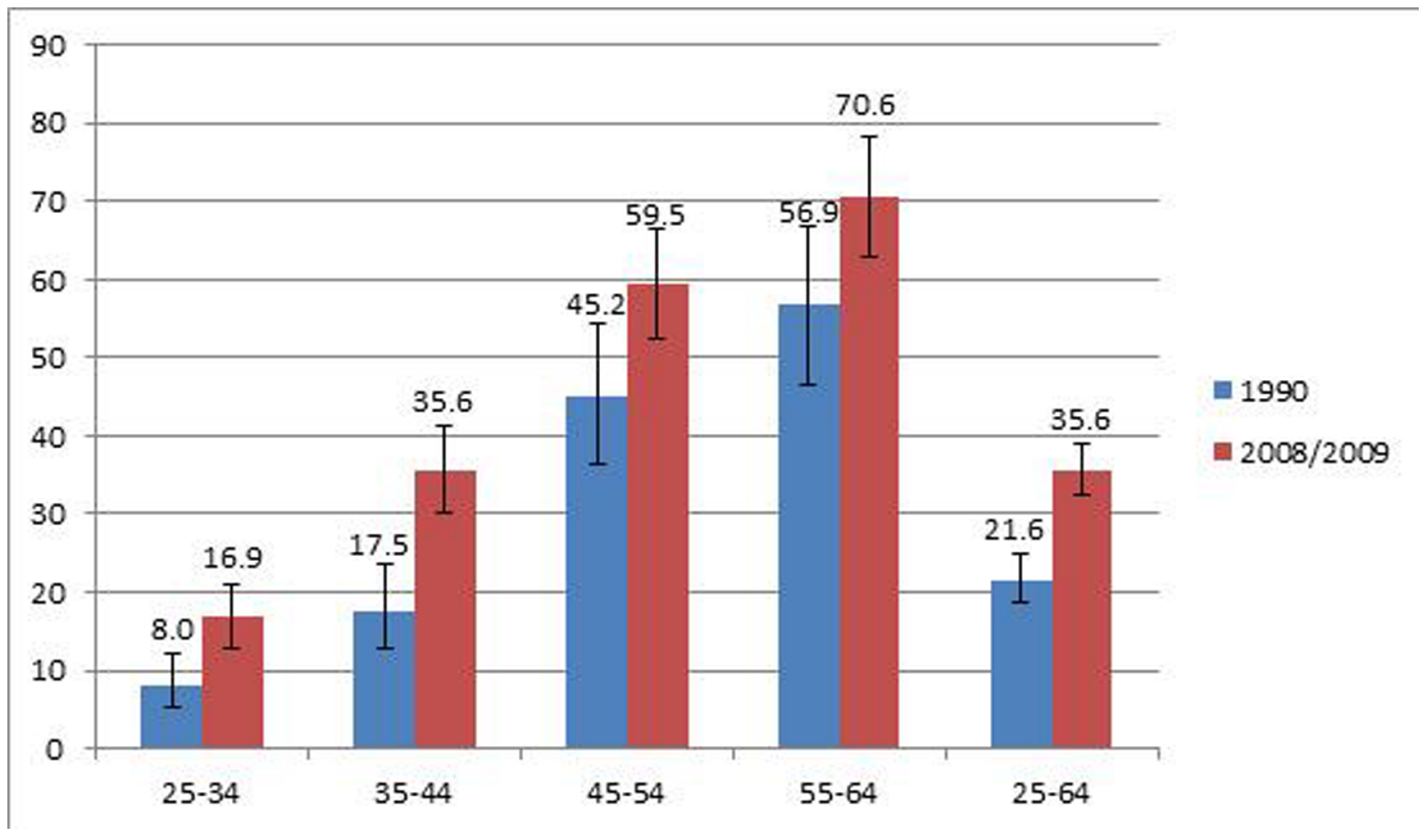
Prevalence (with 95% confidence intervals represented by error bars) of hypertension among 25–64-year-old adults in 1990 and 2008/09 presented by age category.

Among 25–64-year-old participants, the hypertension prevalence was significantly higher in 2008/09 (35.6%, 95% CI: 32.3–39.0) than in 1990 (21.6%, 95% CI: 18.6–24.9) **(** **Figure 3** **)**. By age category, however, it reached significance only in those under 45 years of age.

As presented in **Table 1**, participants with hypertension in 2008/09 were significantly older, less educated, more urbanised, more likely to be pensioners and live in better quality housing such as private units than those without hypertension. However, asset index, an indicator of wealth, did not differ between groups.

**Table 1:** Socio-demographic, psychosocial and cardiovascular disease risk factors presented by hypertension status.

*Socio-demographic factors:*	Hypertension	No hypertension	p-value
Number	461	638	
Age in years, mean ± SD	50.2±11.6	38.3±11.2	<0.001
Gender: men, %	47.5	47.5	0.984
Education: ≤7 years, %	46.2	26.9	<0.001
Employment Status, %:			<0.001
Employed	18.6	25.3	
Unemployed	53.7	65.4	
Pensioners	19.7	4.4	
Other	8.0	4.9	
Housing Type, %:			0.002
Built formal unit (private)	27.2	18.9	
Council/core house/hostel	28.2	25.6	
Informal shack/other	44.6	55.5	
% life in urban area, mean ± SD	65.6±30.5	59.2±33.7	0.001
Asset Index by tertiles, %:			0.177
1^st^ (poorest)	29.7	35.4	
2^nd^	34.1	33.4	
3^rd^ (richest)	36.3	31.2	
***Psychosocial factors/influences:***			
Perceived health status: poor, %	25.6	15.9	<0.001
Sense of coherence: mean ± SD	54.6±10.5	54.5±10.4	0.776
Locus of control: mean ± SD	18.5±2.9	18.9±3.1	0.022
Total adverse life events: mean ± SD	7.7±3.0	7.5±2.8	0.246
Impact within last 6 months	3.7±2.9	4.1±2.9	0.012
Lifetime impact >6 months	10.5±4.3	9.6±4.3	0.001
***Cardiovascular disease risk factors,%***			
Family history of hypertension	43.8	35.9	0.058
Lifestyle/behavioural risk factors:			
Smoke: ≥1cigarette/day	25.0	29.1	0.455
Problematic alcohol use: CAGE ≥2	31.2	34.3	0.318
Moderate to vigorous activity/week: <150 minutes	9.4	4.9	0.006
Anthropometry:			
BMI ≥25 kg/m^2^,	66.9	51.4	<0.001
Raised waist circumference	62.0	50.4	0.001
Raised waist-to-hip ratio	62.0	42.6	<0.001
Dysglycaemia:			
Diabetes	21.3	6.6	<0.001
Impaired glucose tolerance	15.9	7.6	<0.001
Impaired fasting glucose	1.2	1.3	0.904
Dyslipidaemia:			
Total cholesterol >5 mmol/l	34.6	17.8	<0.001
HDL-C <1.2 mmol/l	55.3	64.9	0.002
LDL-C >3 mmol/l	52.2	35.9	<0.001
Triglycerides >1.5 mmol/l	24.8	11.1	<0.001
HDL-C:TC <20%	21.4	14.0	0.002

Mean, SD are reported for the study sample and not adjusted for the population; Other: comprised of homemakers, students and those receiving disability grants; Raised waist circumference (WHO): men >94 cm; women >80 cm; Raised waist-to-hip ratio (WHO): men >1.0; women >0.85; HDL-C: high-density lipoprotein cholesterol; LDL-C: low-density lipoprotein cholesterol; TC: total cholesterol.

Regarding psychosocial influences, those with hypertension had a significantly lower LOC, reflective of greater stress, compared to those without hypertension **(** **Table 1** **)** although SOC scores and the total number of adverse life events experienced were similar in the two groups.

Participants with hypertension, compared to their counterparts, had significantly higher rates of diabetes mellitus, poorer lipid profiles, higher rates of all measures of raised adiposity) and physical inactivity **(** **Table 1** **)**.

Among participants with hypertension, significantly more women compared to men were detected (69.5% vs. 32.7%, p<0.001), treated (55.7% vs. 21.9%, p<0.001) and controlled on treatment (32.4% vs. 10.4%, p<0.001) in 2008/09. The most frequent medication used among hypertensive participants were diuretics (35.5%), angiotensin converting enzyme (ACE) inhibitors (23.5%), calcium channel blockers (15.8%) and β-blockers (7.2%).

**Figure 4:**
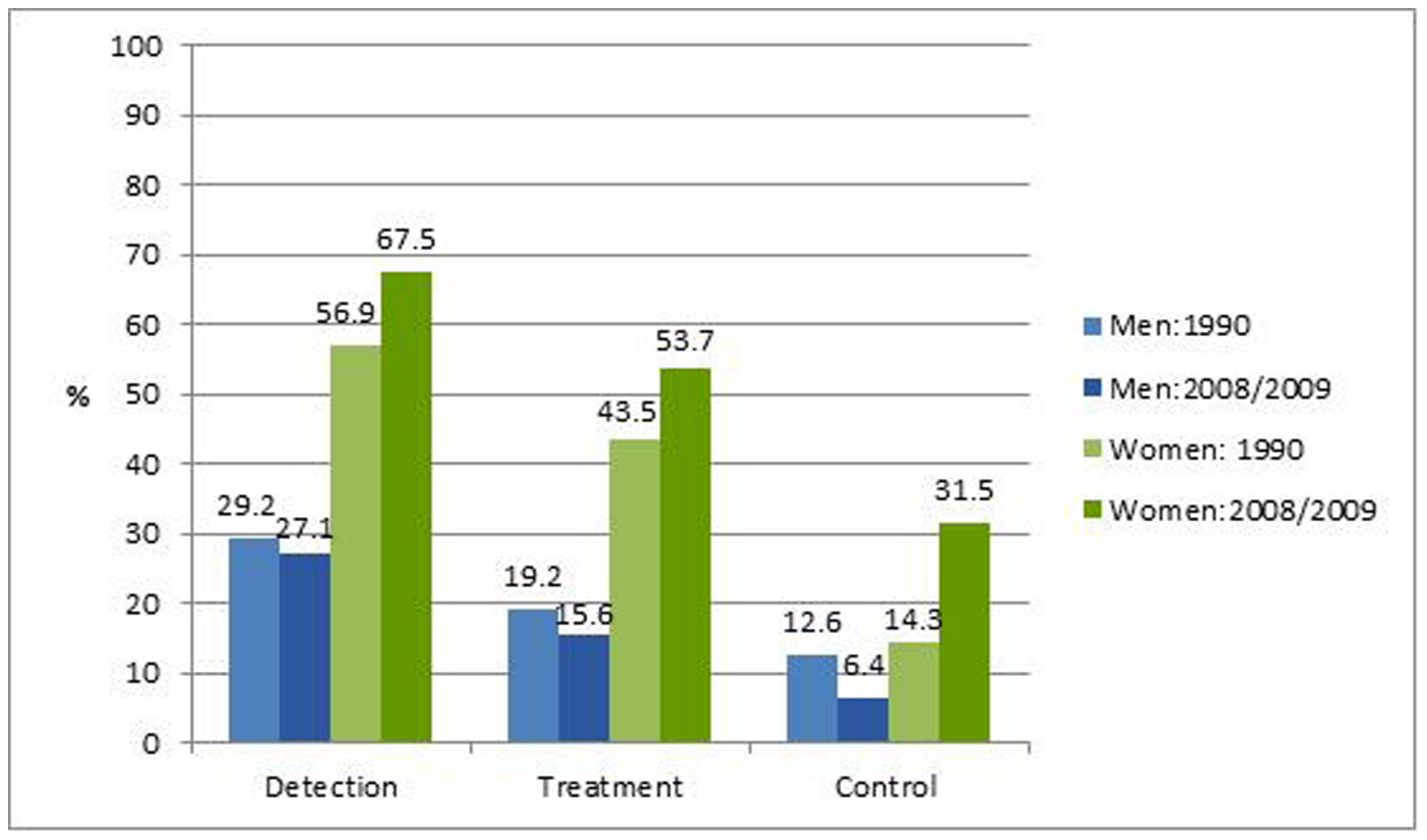
Prevalence of hypertension detection, treatment and control among 25–64-year-old men and women with hypertension in 1990 (n = 166) and 2008/09 (n = 405).

Between 1990 and 2008/09, there was no significant change in the proportion of detected or treated hypertension among 25–64-year-old hypertensive participants although these improved in women and slightly worsened in men **(** **Figure 4** **)**. Among 25–64-year-old men, the prevalence of controlled hypertension decreased, but not significantly, between 1990 (12.6%, 95% CI: 6.3–23.5) and 2008/09 (6.4%, 95% CI: 3.5–11.4). In contrast, the prevalence of controlled hypertension among 25–64-year-old women increased significantly from 14.1% (95% CI: 8.6–22.5) in 1990 to 32.4% (95% CI: 27.1–38.3) in 2008/09.

In the three multiple logistic models for hypertension, each psychosocial variable was entered independently. The model with SOC is shown in **Table 2**. The odds for hypertension increased with older age (>60-year-old vs. ≤30- year-olds: odds ratio (OR), 23.68, 95% CI: 11.57–48.47, p<0.001), a family history of hypertension (OR: 1.49, 95% CI: 1.10–2.03, p = 0.011), higher BMI (OR: 1.04, 95% CI: 1.02–1.07, p = 0.002), problematic drinking (OR: 1.50, 95% CI: 1.05–2.16, p = 0.027), physical inactivity and urbanisation. Surprisingly, higher SOC, an indicator of lower stress, was positively associated with hypertension (OR: 1.02, 95% CI: 1.01–1.04, p = 0.011). When LOC and adverse life events replaced SOC in their respective models, neither was related to hypertension after adjusting for the other factors.

**Table 2:** Multiple logistic regression model for associations with hypertension.

Variable	Hypertension (n = 461)			
	Odds Ratio	95% Confidence Interval		p-value
		Lower limit	Upper limit	
Age category: ≤30 years	1.00			
31–40 years	3.30	1.79	6.07	**<0.001**
41–50 years	7.71	4.13	14.38	**<0.001**
51–60 years	19.51	10.55	36.07	**<0.001**
>60 years	23.68	11.57	48.47	**<0.001**
Sex: female	0.85	0.56	1.30	0.444
Family history of hypertension: yes	1.49	1.10	2.03	**0.011**
Increasing BMI (kg/m^2^)	1.04	1.02	1.07	**0.002**
Moderate to vigorous physical activity:<150 min/week	1.00			
150–500 min/week	0.52	0.31	0.87	**0.012**
500–1000 min/week	0.47	0.25	0.88	**0.018**
>1 000 min/week	0.41	0.24	0.69	**0.001**
% life in city: <50%	1.00			
50–90%	1.62	1.13	2.34	**0.010**
90–100%	1.55	1.04	2.31	**0.033**
Problem drinking : CAGE ≥2	1.50	1.05	2.16	**0.027**
Current smoking : daily/occasionally	0.96	0.61	1.51	0.855
Sense of coherence (SOC): higher	1.02	1.01	1.04	**0.011**
Locus of control (LOC): higher*	0.98	0.93	1.04	0.509
Number of adverse life events: higher*	1.00	0.98	1.02	0.810

* When LOC and adverse life events independently replaced SOC in above model, there were no changes in the direction or significance of the other variables.

## Discussion

These data reveal that hypertension represents an enormous burden in this population with the 38.9% age-standardised prevalence among the highest reported in SSA. An equally high hypertension prevalence was found in Mozambique [25] and Seychelles [26].

In keeping with other SSA studies [6], [27], [28], and the findings from Mozambique and Seychelles, hypertension was likely to be higher among older participants in this study. The positive association between age and hypertension will fuel the hypertension epidemic given demographic shifts towards older age following better medical care and the control of infectious diseases, especially HIV/AIDS.

The significantly higher prevalence of adiposity, diabetes mellitus and dyslipidaemia among participants with hypertension confirms that hypertension rarely occurs in isolation and conforms with other reports [29], [30]. The clustering of other CVD risk factors with hypertension increases the overall risk for CVD [29] and highlights the need to examine for other CVD risk factors in the presence of hypertension.

The significant association between BMI and hypertension accords with other data [6], [26]–[28], [30], including the Seychelles study [31], and is in keeping with the high proportion (68%) of hypertensive disease attributable to BMI ≥21 kg/m^2^ in South Africa in 2000 [32]. Furthermore, the higher mean BMI among 25–64-year-old women in 2008/09 compared to 1990 (33.0 kg/m^2^ vs. 29.5 kg/m^2^) was likely an important contributor to their higher hypertension prevalence in 2008/09. Addressing obesity in South Africa remains a major challenge because its positive connotations make it acceptable and even desirable. African women perceive overweight to be associated with dignity, respect, wealth, strength, attractiveness, happiness and health, and with being well-treated by their husbands [32], [33].

In 25–64-year-old men, however, mean BMI remained unchanged between 1990 (24.3 kg/m^2^) and 2008/09 (23.6 kg/m^2^) suggesting that other hypertensive risk factors probably contributed to their rising hypertension prevalence during this period. Among the complex and multifactorial contributors to hypertension [34], alcohol likely played a role in men in this study. The prevalence of alcohol consumption among men in 2008/09 (68.5%) was higher than in 1990 (56.7%). Additionally, problem drinking was significantly associated with hypertension in 2008/09.

Nonetheless, factors not examined in the current study, such as high salt intake, may also be responsible for the high hypertension prevalence considering that high levels of salt consumption have been recorded in South African black populations [35]. Salt consumption is thought to be the major factor contributing to increasing BP in populations [31] with black individuals reported to be particularly sodium sensitive [31], [34].

Despite the observed protective impact of higher levels of physical activity in this study, in keeping with the well-known inverse association of physical activity with hypertension [36], the reported absolute level of inactivity in this study was very low (6.6%). This was not congruent with previous studies that reported much higher levels of physical inactivity in South Africa (about 50%), some of which used the same GPAQ questionnaire [37], [38]. It is highly unlikely that activity levels would have increased so dramatically and probably reflects over-reporting. There is thus a pressing need for a highly reproducible inexpensive and practical tool to objectively measure physical activity in South Africa and other developing regions.

The relationship between urbanisation and hypertension, consistent with other reports [6], [28], [30], supports the notion that urbanisation is a key driver of the increasing hypertension prevalence [31], [39]. The inevitable increase in urbanisation and the adoption of unhealthy lifestyles in South Africa is likely to lead to further rises in the future hypertension prevalence [28]. This highlights the need to improve awareness of the modifiable risk factors for hypertension and to promote healthy living.

The association of higher SOC, a measure of lower psychosocial stress, with hypertension was unexpected, but its relevance is uncertain in view of the very small odds ratio, despite its statistical significance. Reinforcing the limited relevance of this relationship was the absence of associations between the other measures of psychosocial stress tested (LOC and adverse life events) and hypertension.

Based on the available literature and supported by the current findings hypertension management remains a major public health challenge in South Africa. Yet effective medication makes hypertension one of the most modifiable CVD risk factors and optimal management can significantly reduce the risk of complications and decrease the associated social and medical costs [4], [40].

However, amidst the focus on infectious diseases, particularly HIV/AIDS and tuberculosis, overburdened healthcare services and health professionals in South Africa perceive hypertension and other non-communicable diseases (NCDs) as less urgent. Consequently, the health system response has been limited with these conditions marginalised and receiving little priority [41]. However, in accordance with the recent United Nations resolution that seeks to halt the increasing trends in premature deaths from NCDs, the South African Department of Health has prioritised the management of NCDs, which is a significant step for the management of hypertension and other NCD risk factors [42].

The significant improvement in hypertension control among women between 1990 and 2008/09, in contrast to the findings in men, may be due to the availability of better antihypertensive agents or enhanced compliance in that women may be more likely to accept treatment or lifestyle changes more readily [27]. Also, women are more in contact with healthcare services because of maternal and child health programmes, and display greater health-seeking behaviour resulting in greater chances for BP measurements [25], [27]. Men may also be more likely to be employed or looking for employment and consequently time constrained from attending healthcare clinics during office hours. Workplace hypertension screening and treatment programmes could contribute to better detection and control in men who seldom attend healthcare services. Additionally, it has been hypothesised that men may be less likely to attend healthcare facilities for fear of the diagnosis of life-threatening infectious diseases which are more common now than in 1990. Other social, cultural and economic factors may impair control of hypertension and require further exploration in this population.

Innovative solutions with easier access to healthcare as well as efficient and cost-effective healthcare delivery are needed if hypertension management is to improve in this community. Reports from high-income countries demonstrate that it is possible to achieve better hypertension management. While far from optimal, the NHANES 2003–2006 study in the United States found the prevalence of hypertension detection, treatment and control among ≥20-year-old African-Americans to be 79.5%, 71.8% and 44.6%, respectively [43], in each instance much higher than that found in this study.

### Study Limitations

The study limitations included the following: 1) The cross-sectional design which precluded any conclusions about causal associations between hypertension and the associated factors, 2) The low sample realisation in men (64%) characteristic of epidemiological studies in this country and probably due to their reluctance to participate, particularly for the drawing of blood samples. This necessitated higher sampling weights and a loss of precision. 3) Diagnosing hypertension on BP readings taken on one rather than at least three separate occasions which may have led to some overestimation of the prevalence, 4) The use of self-reported rather than objectively measured ambulation or physical activity, and 5) The lack of comparability of all tools used across both studies, for example, the GPAQ and CAGE questionnaires were implemented only in 2008/09 and not in 1990. The use of more standardised methodologies and reporting could improve the quality of comparative studies and guide future efforts.

### Conclusion

The hypertension burden is high and rising in the urban black population of Cape Town with likely contributors including overweight/obesity, alcohol consumption, urbanisation, physical inactivity and ageing populations, among other factors. However, although hypertension is the single most prevalent CVD risk factor in black South Africans and the predominant contributor to CVD morbidity and mortality [4], [44], this silent epidemic is not matched with comparable levels of appropriate intervention. Hypertension management needs to be prioritised and care provided in the context of a total CVD risk approach. Additionally, population-based strategies to improve diets and encourage greater physical activity are needed [4]; otherwise increasing risk factors may contribute to higher hypertension rates in the future.
